# Country of first birth and neonatal outcomes in migrant and Norwegian-born parous women in Norway: a population-based study

**DOI:** 10.1186/s12913-020-05415-y

**Published:** 2020-06-15

**Authors:** Eline S. Vik, Roy M. Nilsen, Vigdis Aasheim, Rhonda Small, Dag Moster, Erica Schytt

**Affiliations:** 1 Faculty of Health and Social Sciences, Western Norway University of Applied Sciences, Campus Kronstad, Inndalsveien 28, 5063 Bergen, Norway; 2 Department of Global Public Health and Primary Care, University of Bergen, Bergen, Norway; 3 Department of Women’s and Children’s Health, Karolinska Institutet, Stockholm, Sweden; 4 Judith Lumley Centre, La Trobe University, Melbourne, Australia; 5 Department of Pediatrics, Haukeland University Hospital, Bergen, Norway; 6 Centre for Clinical Research Dalarna, Uppsala University, Uppsala, Sweden

**Keywords:** Immigration, Parous women, Neonatal outcomes, Obstetric history, Predictor

## Abstract

**Background:**

This study compares subsequent birth outcomes in migrant women who had already had a child before arriving in Norway with those in migrant women whose first birth occurred in Norway. The aim of this study was to investigate the associations between country of first birth and adverse neonatal outcomes (very preterm birth, moderately preterm birth, post-term birth, small for gestational age, large for gestational age, low Apgar score, stillbirth and neonatal death) in parous migrant and Norwegian-born women.

**Methods:**

National population-based study including second and subsequent singleton births in Norway from 1990 to 2016. Data were retrieved from the Medical Birth Registry of Norway and Statistics Norway. Neonatal outcomes were compared between births to: 1) migrant women with a first birth *before* immigration to Norway (*n* = 30,062) versus those with a first birth *after* immigration (*n* = 66,006), and 2) Norwegian-born women with a first birth *outside* Norway (*n* = 6205) versus those with a first birth *in* Norway (*n* = 514,799). Associations were estimated as crude and adjusted odds ratios (aORs) with 95% confidence intervals (CIs) using multiple logistic regression.

**Results:**

Migrant women with a first birth *before* immigrating to Norway had increased odds of adverse outcomes in subsequent births relative to those with a first birth *after* immigration: very preterm birth (22–31 gestational weeks; aOR = 1.27; CI 1.09–1.48), moderately preterm birth (32–36 gestational weeks; aOR = 1.10; CI 1.02–1.18), post-term birth (≥42 gestational weeks; aOR = 1.19; CI 1.11–1.27), low Apgar score (< 7 at 5 min; aOR = 1.27; CI 1.16–1.39) and stillbirth (aOR = 1.29; CI 1.05–1.58). Similar results were found in the sample of births to Norwegian-born women.

**Conclusions:**

The increased odds of adverse neonatal outcomes for migrant *and* Norwegian-born women who had their first births outside Norway should serve as a reminder of the importance of taking a careful obstetric history in these parous women to ensure appropriate care for their subsequent pregnancies and births in Norway.

## Background

The World Health Organization promotes reducing health inequalities for migrant families [1]. With the growing proportion of migrant women giving birth in high-income countries [1, 2], increased knowledge about their pregnancy outcomes is needed [3]. Migrant women may be of good health, sometimes even better health than the host population; a phenomenon often referred to as the healthy migrant effect [4, 5]. However, increased risk of adverse pregnancy outcomes including preterm birth [6, 7] and perinatal mortality [8] have been reported for refugees in particular.

Nearly half of women giving birth in high income countries are parous [9] and maternity care is mainly tailored to the host population with particular focus on first-time mothers and those with a complicated first pregnancy and childbirth [10]. We have previously reported that migrant women who gave birth to their first baby *before* immigration to Norway had an increased risk of stillbirth in later births compared with migrant women who gave birth to their first baby in Norway [11]. In the current study, we explore whether this increased risk applies also to other adverse neonatal outcomes, and whether the findings are unique to migrant women or if they also apply to Norwegian-born women who return to Norway after a first childbirth abroad.

The aim was to investigate the associations between country of first birth and adverse neonatal outcomes (very preterm birth, moderately preterm birth, post-term birth, small for gestational age, large for gestational age, low Apgar score, stillbirth and neonatal death) in parous migrant and Norwegian-born women in Norway.

## Methods

### Study design

In this national population-based study, we used individual record data from the Medical Birth Registry of Norway (MBRN) [12, 13] and Statistics Norway (SSB) [14]. The data were merged using each woman’s unique national identity number. The MBRN is the repository for mandatory notification of all births in Norway, and includes information on women’s obstetric background, maternal health before and during pregnancy, current pregnancy, labour and birth, and maternal and infant outcomes. The MBRN data are collected from medical records and women’s self-reported obstetric history. SSB provides information on migration and socioeconomic factors.

### Setting

In Norway, the health care system is considered of high quality with low maternal and child mortality rates [15]. All women are entitled to free maternity care in Norway, and the vast majority of women give birth in public hospitals (99%) [16]. Unless there are medical complications necessitating specialist obstetric care, women may choose antenatal care provided by either a general practitioner, a midwife, or a combination of the two [17]. However, inequalities in health care have been reported and migrant women in Norway appear more likely to receive suboptimal care compared to non-migrant women [18]. In 2018, 29% of children born in Norway were born to a migrant mother [19].

### Study population

**Fig. 1:**
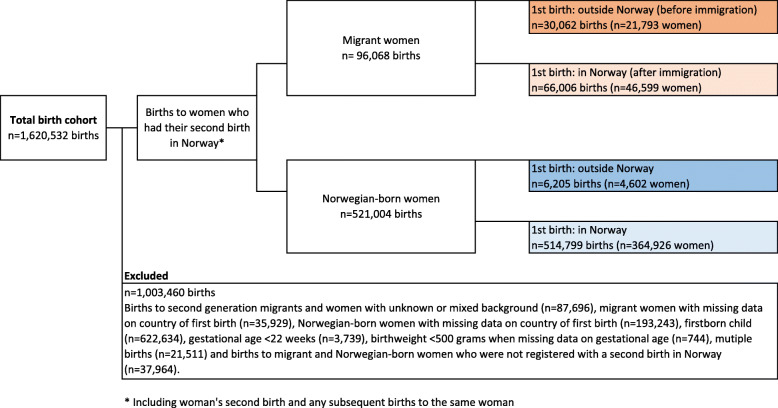
Flowchart of the derivation of the study sample (*n* = 1,620,532). * Including woman’s second birth and any subsequent births to the same woman

The main goal of this study was to compare subsequent birth outcomes in migrant women who already had a child before arriving in Norway (defined as the exposure group) with the same outcomes in migrant women with a first birth in Norway (defined as the comparison group). In order to control for possible parity-related differences between exposure and comparison groups, we restricted the exposure group to include women with only one birth before arriving in Norway (Fig. 1).

Initially, MBRN comprised 1,620,532 births during the period 1990–2016. Births to second generation migrant women, those with unknown or mixed background, such as adoptees or women with one Norwegian-born and one foreign-born parent, were excluded (*n* = 87,696). The final sample included the second and any subsequent singleton births to foreign-born women with two foreign-born parents (*n* = 96,068 births to migrant women), and Norwegian-born women with two Norwegian-born parents (*n* = 521,004 births to Norwegian-born women) giving birth in Norway between the years 1990 and 2016 (Fig. 1).

### Country of woman’s first birth

To derive information on whether a woman had a first child before or after immigration to Norway, we used the following algorithms:

#### Migrant women

The country of a woman’s first birth was determined by the woman’s first parity registered in the MBRN dataset. If a parous woman’s first birth was in the dataset, the birthplace of her firstborn baby was classified as Norway. If the woman’s first birth was not in the dataset, the birthplace was classified as other than Norway. Women with permission to stay in Norway prior to 1990 may or may not have given birth in Norway before 1990 (the study period commencement) and were therefore excluded (*n* = 35,929).

#### Norwegian-born women

To identify country of first birth we excluded births to any woman 13 years or older in 1990 whose first birth was not available in the MBRN dataset (*n* = 193,243) and therefore could in theory have had previous babies before 1990. The women’s first parity registered in the dataset was then used to identify country of first birth in the Norwegian-born women. The age limit was chosen based on the fact that the youngest mothers in our dataset were 13 years of age.

### Adverse neonatal outcomes

Gestational age was based on ultrasound estimation or, when such information was lacking, calculated from the first day of the last menstrual period. Very preterm birth, moderately preterm and post-term birth were defined as births in gestational week 22–31, 32–36 and > 42, respectively. In the analyses of very preterm birth, moderately preterm and post-term birth, we excluded births with unknown gestational age (migrant women *n* = 1512; Norwegian-born women *n* = 12,677) and term births were used as comparison group. In the analyses of small for gestational age (SGA) and large for gestational age (LGA) we also excluded births with unknown birthweight (migrant women *n* = 63; Norwegian-born women *n* = 403). For calculating SGA and LGA, we used Norwegian standards combining information on gestational age, birthweight and gender [20]. Low Apgar Score was defined as < 7 at 5 min. Stillbirth was defined as a pregnancy loss at ≥22 weeks of gestation or birthweight ≥500 g if data on gestational age were missing. Neonatal death was defined as a live born infant at ≥22 weeks of gestation (or with a birthweight ≥500 g if data on gestational age was missing) who died within 28 days after the birth.

### Other variables

From the MBRN, we also obtained data on year of birth, maternal age (< 25, 25–34, ≥35 years), single status (yes, no), parity (1, 2, 3, ≥4), smoking in early pregnancy (yes/no) and previous stillbirth (yes, no).

For each birth year, SSB provided data on maternal level of education (no education, primary school, secondary school, university/college, missing), mother’s gross income (categorized into quartiles, missing), reason for immigration (Nordic migrants, work/education, family reunion or establishment, refugee, missing), and paternal origin (Norwegian-born, foreign-born, missing). Maternal country of birth from SSB was used to classify women according to seven Global Burden of Disease super regions (GBD) [21]: High income countries; Central Europe, Eastern Europe, and Central Asia; Sub-Saharan Africa; North Africa and Middle East; South Asia; Southeast Asia, East Asia, and Oceania; Latin America and Caribbean. Maternal length of residence was calculated as the difference between the year of birth and the year a woman officially received her permission to stay in Norway (< 2 years, 2–5 years, 6–9 years, ≥10 years). Maternal age at immigration was calculated as the difference between maternal age at birth and her length of residence (< 18 years, ≥18 years).

### Statistics

Neonatal outcomes were compared between births to: 1) migrant women with a first birth *before* immigration to Norway versus those with a first birth *after* immigration, and 2) Norwegian-born women with a first birth *outside* Norway versus those with a first birth *in* Norway. We also compared births to migrant women with a first birth *before* immigration to Norway versus Norwegian-born women with a first birth *outside* Norway.

Logistic regression analyses were used to investigate possible associations between country of first childbirth (Norway/Other than Norway) and adverse neonatal outcomes in subsequent births. Associations were reported as odds ratios with 95% confidence intervals. Adjustment variables were year of birth, maternal age, parity, marital status, maternal education and mother’s gross income. To account for dependency between births by the same mother, we used robust standard errors that allowed for within-mother clustering.

To avoid list-wise deletion and potential bias due to missing data in covariates in the adjusted regression models, we used a multiple imputation technique to replace missing values in covariates. Ten imputed datasets were created using the multivariate normal model [22]. Separate imputation models were created for each neonatal outcome and included the respective outcome (very preterm birth, moderately preterm birth, post-term birth, SGA, LGA, low Apgar score, stillbirth or neonatal death), as well as country of first childbirth and adjustment variables.

Analyses were performed using Stata IC version 16 (Stata Statistical Software, College Station, TX, USA) for Windows.

## Results

Table 1 shows the background characteristics of the four groups at the time of the woman’s second birth. Compared to migrant women with a first birth in Norway, migrant women with a first birth *before* immigration to Norway had more often missing data on education, lower or missing data on income. They also reported higher smoking prevalence in early pregnancy, a higher rate of previous stillbirth, they were more often from *Central Europe, Eastern Europe & Central Asia*, shorter length of residence in Norway, higher age at migration, a foreign-born father to the baby, or missing information on paternal origin. Further, they were less likely to originate from *High income countries* or *North Africa & Middle East*. Compared with Norwegian-born women with a first birth in Norway, Norwegian-born women with a first birth *outside* Norway were more likely to: be younger, be of single status, have lower levels of education, have higher income, smoke in early pregnancy, have experienced a previous stillbirth, report a foreign-born father to the baby, or have missing information on paternal origin.

**Table 1:** Background characteristics at the time point for 2nd birth; migrant (*n* = 68,392) and Norwegian-born women (*n* = 369,528)^a^

	Migrant women’s first birth				Norwegian-born women’s first birth			
	Before immigration		After immigration		Outside Norway		In Norway	
	n	%	n	%	n	%	n	%
Total	21,793	31.9	46,599	68.1	4602	1.2	364,926	98.8
Age (years)								
< 25	3027	13.9	6631	14.2	1419	30.8	46,724	12.8
25–34	14,535	66.7	31,949	68.6	2991	65.0	267,908	73.4
≥ 35	4231	19.4	8019	17.2	192	4.2	50,294	13.8
Single status^b^	1365	6.3	3073	6.6	442	9.6	16,899	4.6
Mother’s education								
No education	367	2.7	653	1.8	0	0.0	3	0.0
Primary education	3889	28.3	10,275	28.3	1112	24.2	58,473	16.0
Secondary school	3518	25.6	9244	25.4	1451	31.6	135,373	37.1
University/college	5985	43.5	16,188	44.5	2023	44.1	170,715	46.8
Mother’s education, missing	8034	36.9	10,239	22.0	16	0.4	362	0.1
Mother’s income								
≤ 25 percentile	5194	41.9	9386	26.8	692	15.9	61,779	18.0
25–50 percentile	1971	15.9	5981	17.1	674	15.5	83,609	24.3
50–75 percentile	2784	22.4	8838	25.2	1143	26.3	98,455	28.6
≥ 75 percentile	2455	19.8	10,839	30.9	1839	42.3	100,274	29.1
Mother’s income, missing	9389	43.1	11,555	24.8	254	5.5	20,809	5.7
Smoking in early pregnancy^c^	1203	7.8	1611	4.7	709	17.7	32,810	14.0
Previous stillbirth	214	1.2	235	0.6	77	1.8	1075	0.4
**Migration**								
Maternal origin (GBD)								
High income country	3864	17.7	10,266	22.0	4602	100.0	364,926	100.0
Central Europe, Eastern Europe & Central Asia	7488	34.4	11,076	23.8				
Sub-Saharan Africa	2714	12.5	5491	11.8				
North Africa & Middle East	2482	11.4	7797	16.7				
South Asia	873	4.0	3208	6.9				
Southeast Asia, East Asia & Oceania	3625	16.6	7516	16.1				
Latin America & Caribbean	747	3.4	1245	2.7				
Reason for immigration								
Nordic migrants	1720	8.0	5514	12.0				
Work/education	3170	14.8	7960	17.3				
Family reunion/establishment	12,789	59.5	25,338	55.1				
Refugee	3817	17.8	7137	15.5				
Reason for immigration, missing	297	1.4	650	1.4				
Length of Residence								
< 2 years	10,659	48.9	1801	3.9				
2–5 years	8618	39.5	22,952	49.3				
6–9 years	1751	8.0	13,116	28.2				
≥ 10 years	765	3.5	8730	18.7				
Age at migration < 18 years	367	1.7	5231	11.2				
Foreign-born father	13,359	81.7	29,094	64.7	594	13.3	21,058	5.8
Paternal origin, missing	5431	24.9	1636	3.5	148	3.2	3282	0.9

^a^Percentages are calculated from non-missing data if not otherwise noted

^b^Includes unmarried, single, divorced, separated, widowed and other/missing.

^c^Data on smoking from 1999 onwards

**Fig. 2:**
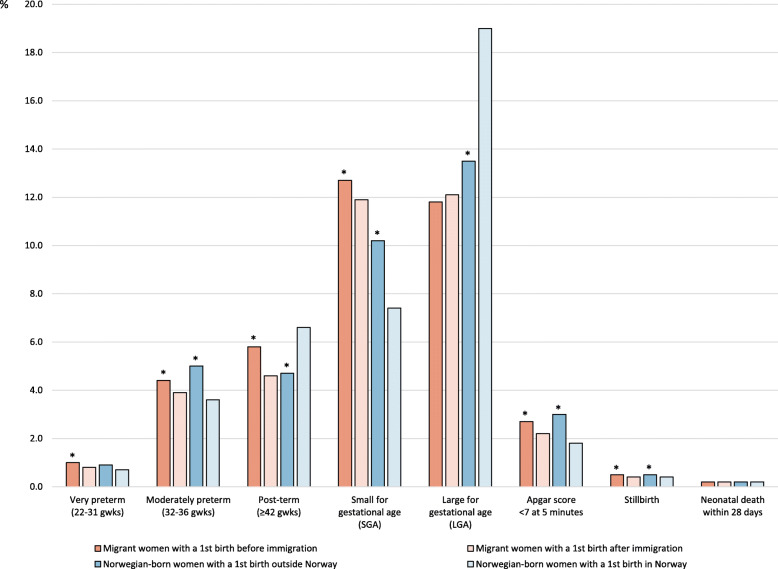
Prevalence of adverse neonatal outcomes in second and subsequent births in migrant and Norwegian-born women (1990–2016). * *p*-values < 0.05, when comparing birth outcomes in either the two groups of migrant women or the two groups of Norwegian-born women

The prevalence of adverse neonatal outcomes in second and subsequent births to migrant and Norwegian-born women in relation to country of first birth is shown in Fig. 2. The prevalence of most adverse outcomes was slightly higher in births to migrant women with a first birth before immigration to Norway compared to those with a first birth after immigration: very preterm birth (1.0% vs 0.8%; *p* < 0.001), moderately preterm birth (4.4% vs 3.9%; *p* < 0.001), post-term birth (5.8% vs 4.6%; *p* < 0.001), SGA (12.7% vs 11.9%; *p* < 0.001), low Apgar score (2.7% vs 2.2%; *p* < 0.001), and stillbirth (0.5% vs 0.4%; *p* < 0.01). For the migrant women the prevalence of LGA (11.8% vs 12.1%; *p* = 0.178) and neonatal death (0.2% vs 0.2%; *p* = 0.988) was similar in both groups.

Compared to those with a first birth in Norway (Fig. 2), Norwegian-born women with a first birth outside Norway had higher prevalence of moderately preterm birth (5.0% vs 3.6%; *p* < 0.001), SGA (10.2%vs 7.4%; *p* < 0.001), low Apgar score (3.0% vs 1.8%; *p* < 0.001) and stillbirth (0.5% vs 0.4%; *p* < 0.05), and lower prevalence of post-term birth (4.7% vs 6.6%; *p* < 0.001) and LGA (13.5% vs 19.0%; *p* < 0.001). For the Norwegian-born women, the prevalence of very preterm birth (0.9% vs 0.7%; *p* = 0.141) neonatal death (0.2% vs 0.2%; *p* = 0.472) was similar in both groups.

In second and subsequent births to migrant and Norwegian-born women the prevalence of SGA was higher, and LGA lower, if the father of the baby was foreign-born compared to births where the father was Norwegian-born (SGA: 13.3% vs 8.7%; *p* < 0.001 and 8.5% vs 7.3%; *p* < 0.001; LGA: 10.8% vs 15.1%; *p* < 0.001 and 16.5% vs 19.1%; *p* < 0.001, respectively) (not shown).

The crude and adjusted associations between *migrant* women’s country of first birth and adverse neonatal outcomes are shown in Table 2. After adjustments for year of birth, parity, maternal age, marital status, maternal education and income, analyses show that women who gave birth to their first baby before immigrating to Norway had increased odds of very preterm birth (aOR = 1.27; CI 1.09–1.48), moderately preterm birth (aOR = 1.10; CI 1.02–1.18), post-term birth (aOR = 1.19; CI 1.11–1.27), low Apgar score (aOR = 1.27; CI 1.16–1.39) and stillbirth (aOR = 1.29; CI 1.05–1.58) compared to foreign-born women who had their first baby after immigrating to Norway. The results were similar when women from high-income countries were excluded from the analyses (data not shown).

**Table 2:** Associations between migrant women’s country of first birth and adverse neonatal outcomes (1990–2016)

	n births	n cases	Crude OR	Adjusted OR	Adjusted OR	Adjusted OR
Adverse neonatal outcomes			(95% CI)	(95% CI)*	(95% CI)†	(95% CI) ‡
Very preterm (22–31 weeks)§						
Norway	62,366	532	1.00	1.00	1.00	1.00
Other	27,965	308	1.29 (1.12–1.50)	1.26 (1.09–1.47)	1.26 (1.09–1.46)	1.27 (1.09–1.48)
Moderately preterm (32–36 weeks)§						
Norway	64,348	2514	1.00	1.00	1.00	1.00
Other	28,938	1281	1.14 (1.06–1.22)	1.11 (1.03–1.19)	1.11 (1.03–1.19)	1.10 (1.02–1.18)
Post-term (≥42 weeks)§						
Norway	62,096	2994	1.00	1.00	1.00	1.00
Other	27,825	1701	1.29 (1.20–1.37)	1.21 (1.13–1.29)	1.20 (1.13–1.29)	1.19 (1.12–1.27)
Small for gestational age (SGA)						
Norway	65,092	7738	1.00	1.00	1.00	1.00
Other	29,401	3743	1.08 (1.03–1.13)	1.07 (1.02–1.12)	1.07 (1.02–1.12)	1.05 (1.00–1.10)
Large for gestational age (LGA)						
Norway	65,092	7847	1.00	1.00	1.00	1.00
Other	29,401	3454	0.97 (0.93–1.02)	0.97 (0.92–1.01)	0.97 (0.93–1.02)	0.98 (0.93–1.03)
Apgar score < 7 at 5 min						
Norway	66,006	1418	1.00	1.00	1.00	1.00
Other	30,062	824	1.28 (1.18–1.40)	1.28 (1.17–1.40)	1.27 (1.16–1.39)	1.27 (1.16–1.39)
Stillbirth						
Norway	66,006	261	1.00	1.00	1.00	1.00
Other	30,062	157	1.32 (1.08–1.62)	1.29 (1.06–1.58)	1.29 (1.05–1.59)	1.29 (1.05–1.58)
Neonatal death within 28 days						
Norway	66,006	138	1.00	1.00	1.00	1.00
Other	30,062	63	1.00 (0.74–1.36)	0.96 (0.71–1.30)	0.96 (0.70–1.30)	0.95 (0.69–1.30)

* Adjusted for year of birth, parity, maternal age and marital status

† Adjusted for * and maternal education

‡ Adjusted for *, † and mother’s gross income

§ Weeks of gestation; term births were used as comparison group

The crude and adjusted associations between *Norwegian-born* women’s country of first birth and adverse neonatal outcomes are shown in Table 3. The adjusted analyses show increased odds of very preterm birth (aOR = 1.32; 1.00–1.73), moderately preterm birth (aOR = 1.36; CI 1.19–1.55), post-term birth (aOR = 1.23; CI 1.08–1.40), SGA (aOR = 1.43; CI 1.31–1.57), low Apgar score (aOR = 1.61; CI 1.38–1.88) and stillbirth (aOR = 1.69; CI 1.18–2.42), and decreased odds for LGA (aOR = 0.74; CI 0.68–0.80) in Norwegian-born women with a first birth outside Norway, compared to Norwegian-born women with a first birth in Norway.

**Table 3:** Associations between Norwegian-born women’s country of first birth and adverse neonatal outcomes (1990–2016)

	n births	n cases	Crude OR	Adjusted OR	Adjusted OR	Adjusted OR
Adverse neonatal outcomes			(95% CI)	(95% CI)*	(95% CI) †	(95% CI) ‡
Very preterm (22–31 weeks)§						
Norway	480,589	3592	1.00	1.00	1.00	1.00
Other	5865	54	1.23 (0.94–1.62)	1.32 (1.01–1.74)	1.31 (1.00–1.72)	1.32 (1.00–1.73)
Moderately preterm (32–36 weeks)§						
Norway	495,060	18,063	1.00	1.00	1.00	1.00
Other	6117	306	1.39 (1.22–1.58)	1.37 (1.20–1.57)	1.36 (1.19–1.55)	1.36 (1.19–1.55)
Post-term (≥42 weeks)§						
Norway	480,497	33,033	1.00	1.00	1.00	1.00
Other	5815	291	0.71 (0.63–0.81)	1.23 (1.08–1.40)	1.23 (1.08–1.40)	1.23 (1.08–1.40)
Small for gestational age (SGA)						
Norway	501,753	37,174	1.00	1.00	1.00	1.00
Other	6171	632	1.43 (1.30–1.56)	1.45 (1.33–1.59)	1.44 (1.31–1.57)	1.43 (1.31–1.57)
Large for gestational age (LGA)						
Norway	501,753	95,058	1.00	1.00	1.00	1.00
Other	6171	834	0.67 (0.62–0.73)	0.73 (0.67–0.80)	0.74 (0.68–0.80)	0.74 (0.68–0.80)
Apgar score < 7 at 5 min						
Norway	514,799	9279	1.00	1.00	1.00	1.00
Other	6205	187	1.69 (1.46–1.97)	1.62 (1.39–1.89)	1.61 (1.39–1.88)	1.61 (1.38–1.88)
Stillbirth						
Norway	514,799	1789	1.00	1.00	1.00	1.00
Other	6205	31	1.44 (1.01–2.05)	1.69 (1.18–2.42)	1.67 (1.17–2.40)	1.69 (1.18–2.42)
Neonatal death within 28 days						
Norway	514,799	808	1.00	1.00	1.00	1.00
Other	6205	12	1.23 (0.70–2.18)	1.59 (0.89–2.83)	1.58 (0.89–2.81)	1.59 (0.89–2.83)

* Adjusted for year of birth, parity, maternal age and marital status

† Adjusted for * and maternal education

‡ Adjusted for *, † and mother’s gross income

§ Weeks of gestation; term births were used as comparison group

Finally, we compared the outcomes for migrants and Norwegian-born women who all had had their first birth outside Norway. After adjustments for year of birth, parity, maternal age, marital status, maternal education and income, migrant women had increased odds for SGA (aOR = 1.18; CI 1.06–1.32), and decreased odds of moderately preterm birth (aOR = 0.72; CI 0.62–0.85), LGA (aOR = 0.84; CI 0.75–0.93) and low Apgar score (aOR = 0.81; CI 0.67–0.98), relative to Norwegian-born women with a first birth outside Norway.

## Discussion

Migrant women with a first birth *before* immigration to Norway were more likely to experience adverse neonatal outcomes in subsequent births in Norway when compared to migrant women with a first birth *after* immigration. Likewise, Norwegian-born women with a first birth *outside* Norway had increased risk for adverse neonatal outcomes in later births when compared to Norwegian-born women with a first birth *in* Norway.

To our knowledge, this is the first study to investigate a number of adverse neonatal outcomes in subsequent births after a first birth before immigrating to a new country. A first birth before immigration to Norway was associated with increased odds of very preterm, moderately preterm and post-term birth, low Apgar score and stillbirth. Even if the individual’s risk for these adverse neonatal outcomes is small, the conditions are severe with consequences for the family [23] and high costs for society, such as neonatal intensive care and long-term complex health needs [24].

The higher odds of adverse outcomes in migrant women with a first birth before immigration may partly be attributed to the stress of migration. Maternal stress during pregnancy has been identified as an independent risk factor for preterm birth [25], also specific for refugee women [7]. Migrating with children may add to the stress of migration [26, 27], and some women may struggle with feelings of loss or regret after leaving older child(ren) behind [28–30]. Further, near half the women who had given birth before immigration had been in Norway for less than 2 years when their second child was born. These women may lack familiarity with the health care system [31, 32], struggle with language barriers [31] or make suboptimal use of the services [33–35]. Some migrant women also delay their first antenatal visit [34–36], making it difficult to collect a thorough obstetric history. Migrant women are also a heterogeneous group arriving from different countries for a variety of reasons and with different socioeconomic and cultural backgrounds, thus the findings in this study may not apply to all migrant women with a first birth before immigration. Recognizing the complexity of migration is crucial when addressing the various needs of migrant women in maternity care [37].

Somewhat surprisingly, the results related to Norwegian-born women were similar to the ones in the migrant population. A lack of access to information about obstetric history may therefore explain some of the negative outcomes in women with a first birth before immigration. Less attention is often given to parous compared to nulliparous women in antenatal care [10], and health care providers may have less access to previous medical records [31]. Hence, the needs of both migrant and Norwegian-born parous women returning after a first birth abroad may currently be inadequately addressed. Interpretation of the differences between migrant and Norwegian-born women must be made cautiously however, as although we know that migrant women immigrated for a range of reasons, including fleeing war and conflict, we lacked information on the reasons for spending time abroad in the Norwegian-born sample. An alternative explanation for the increased risk of adverse outcomes in the Norwegian-born sample may be that Norwegian-born women who had experienced adverse birth outcomes abroad returned home before their next birth. In our sample, having experienced a previous stillbirth was more common in the Norwegian-born sample of women with a first birth outside Norway compared to Norwegian-born women who had not given birth abroad.

Both migrant and Norwegian-born women with a first birth outside Norway were more likely to report a foreign-born father to the baby compared to women who gave birth to their first child in Norway, and a foreign-born father was associated with an increased prevalence of SGA and a decreased prevalence of LGA in our material. The differences in birthweight between migrant and non-migrant women are difficult to interpret [38, 39]. Such differences may be attributed to normal biological variation as paternal factors can influence fetal growth [40, 41]. However, differences may also reflect maternal and infant health problems or suboptimal care, as infants may be growth-restricted for a variety of reasons [41]. A critical review on birthweight in immigrant populations concludes that birthweight alone is not enough to inform clinical decisions and newborn size charts should serve as screening rather than diagnostic tools [38]. The associations between a foreign-born father and adverse neonatal outcomes need further investigation.

The main strengths of this study include the large sample size and long timespan of the study allowing us to follow the same mothers and their pregnancy outcomes over time (26 years). The standardized collection of data on adverse neonatal outcomes, and the selection of available covariates adjusted for in the regression analyses, add to the strengths of the study. The differences in background characteristics in the Norwegian-born sample are mainly a result of the age limit set to determine country of first birth in these women, and this may limit the conclusions that can be drawn from the Norwegian-born sample. Additionally, we cannot rule out misclassification of self-reported parity. Finally, the low prevalence of adverse outcomes in both migrant and Norwegian-born women limited us from determining if the increased risk of adverse neonatal outcomes was primarily related to the first birth after arriving in Norway or if it also applied to later births to the same mother.

## Conclusions

Both migrant and Norwegian-born women had increased odds of adverse neonatal outcomes in subsequent births if they had their first baby outside Norway compared with if they had their first baby in Norway. The results of this study should serve as a reminder of the importance of collecting a thorough obstetric history from parous women who migrate to a new country after their first birth.

## Data Availability

The data that support the findings of this study are available from the Medical Birth Registry of Norway and Statistics Norway but restrictions apply to the availability of these data, which were used under license for the current study, and so are not publicly available. Data are however available from the authors upon reasonable request and with permission of the Medical Birth Registry of Norway and Statistics Norway.

## Abbreviations

aOR: Adjusted odds ratio
CI: Confidence interval
MBRN: Medical Birth Registry of Norway
OR: Odds ratio
SSB: Statistics Norway
